# Current Standards and Practices Within the Therapy Dog Industry: Results of a Representative Survey of United States Therapy Dog Organizations

**DOI:** 10.3389/fvets.2020.00035

**Published:** 2020-02-07

**Authors:** James A. Serpell, Katherine A. Kruger, Lisa M. Freeman, James A. Griffin, Zenithson Y. Ng

**Affiliations:** ^1^School of Veterinary Medicine, University of Pennsylvania, Philadelphia, PA, United States; ^2^Cummings School of Veterinary Medicine, Tufts University, North Grafton, MA, United States; ^3^National Institutes of Health, Eunice Kennedy Shriver National Institute of Child Health and Human Development, Bethesda, MD, United States; ^4^College of Veterinary Medicine, University of Tennessee, Knoxville, TN, United States

**Keywords:** therapy dog, animal-assisted therapy, animal-assisted interventions, animal welfare, infection control, safety

## Abstract

Organizations that deliver animal-assisted interventions (AAIs), as well as those that train, evaluate, and register therapy dogs, have proliferated in recent decades in the United States (U.S.). Each of these organizations has its own policies and procedures for screening, evaluating, and instructing dogs and their owners/handlers, but little is currently known about the range of different practices that exist nationwide. The aim of this project was to survey a representative, national sample of U.S. therapy dog organizations to investigate commonalities and differences in the types of practices in current use and to compare these to recommendations in existing published guidelines. The findings suggest the need for further research, and highlight a number of areas relating to dog welfare, human safety, and infection control in which many organizations were inconsistent in their adherence to existing guidelines. Of particular concern with regard to animal welfare was the finding that approximately half of the organizations surveyed imposed no time limit on the length of visits. Also, given the potential for zoonotic disease transmission, the finding that only a small minority of organizations prohibit the feeding of raw meat diets and treats to visiting dogs is concerning. This information will help to raise awareness among facilities with therapy animal programs and assist in the development of future best practices within the therapy dog industry.

## Introduction

Organizations that deliver animal-assisted activities and interventions (AAAs, AAIs), as well as those that train, evaluate, and register therapy dogs, have proliferated in recent decades in the United States (U.S.) (1). The American Kennel Club (AKC) currently recognizes 180 different therapy dog organizations in the U.S. (2) and, while the total number of such organizations is unknown, it is likely to exceed this figure substantially. Although several sets of health and safety guidelines have been developed for healthcare facilities planning to implement AAI programs (3–8), the therapy animal “industry” itself is largely self-regulated and there is no nationally-recognized accrediting agency, nor commonly accepted standards or policies, governing their activities (9–11). Many such organizations have their own policies and procedures for screening, evaluating, and instructing dogs and their owners/handlers. However, relatively little is known about the range of different practices that exist nationwide, or whether these practices are adequate to ensure the health, safety, and welfare of both AAI recipients and participating animals and their handlers. Health and safety risks to human patients/clients include transmission of zoonotic disease, bites and scratches, animal-related allergies and accidents, and animal fears/phobias (11), while the risks to the animals primarily involve the potential for overwork and social stress due to excessive or inappropriate interaction with unfamiliar humans (12). Only one previous study investigated health and safety policies in a sample of 27 U.S. therapy animal organizations, and detected significant omissions that placed patients and residents at potential risk of harm (11). Additional work is therefore needed to replicate these findings and identify other potential sources of risk within the therapy animal industry.

A further reason for assessing variation in policy and practice among therapy dog organizations relates to the reproducibility of research on animal-assisted interventions (AAI). Published studies in this area often fail to report the source or background of the dog-handler teams that participate in research studies, thereby making it impossible to determine what standards or criteria, if any, were used to select and train the dogs and/or their handlers (13). Without this type of information, such studies cannot be accurately replicated or their findings independently verified. In addition, having detailed information is critical for funding agencies in order to fully understand the study design and the potential health and safety risks for subjects and the therapy animals themselves. The description and classification of existing standards and practices among therapy dog organizations is therefore an important first step toward the accurate scientific reporting of AAI research methods.

Previous research has focused mainly on health, safety, and infection control practices and policies in healthcare facilities with existing AAI programs. The primary goal of the current project was to survey a representative national sample of U.S. therapy dog organizations to investigate and describe the range of different policies and practices in current use among these organizations, and to identify where these may fall short of what would generally be considered best practice. In addition, six of the largest, national or multiregional therapy animal organizations were invited to participate in the survey for comparative purposes, as well as to assess the level of consensus on policies and practices among the industry leaders. The current study focuses exclusively on dogs, although AAIs are also conducted with a variety of animal species, including horses, cats, and rabbits.

## Methods

### Sample Selection

Two different groups of organizations were invited to complete the survey. Group 1 comprised six prominent national or multiregional therapy animal certification organizations in the U.S.^1^. These organizations certify a large proportion of the therapy animals working in the U.S. and were included primarily for comparative purposes, and to assess the level of consensus on policies and practices among the “industry” leaders.

Group 2 organizations were selected on the basis of the following inclusion and exclusion criteria.

### Inclusion Criteria

- Must perform AAA visits.
- Must have multiple volunteer teams.
- Must have a “basic” program in which volunteers perform AAA visits without treatment goals and without higher-level specialty training.

### Exclusion Criteria

- Is an official affiliate of one of the six largest and/or most prominent therapy dog certifying organizations in the U.S. (e.g., Group 1 organizations) for its basic visitation program.
- States that all of its handlers must be registered with a single therapy dog certifying organization (e.g., “All of our teams are registered with Pet Partners”)^2^.
- Facilities with an in-house therapy dog program (e.g., a hospital where all volunteers only visit patients within that facility).
- An individual handler or therapy dog (e.g., a psychologist working with her dog in private practice).
- Only offers programs with species other than dogs.
- Evaluates dogs for therapy work, but does not have a visiting program.
- Trains dogs for therapy work, but does not have a visiting program.
- Programs that visit using shelter pets.
- All handlers/dogs in the program receive higher-level specialty training (e.g., all teams are trained in crisis response).

### Geographic Selection and Randomization (Group 2)

**Figure 1:**
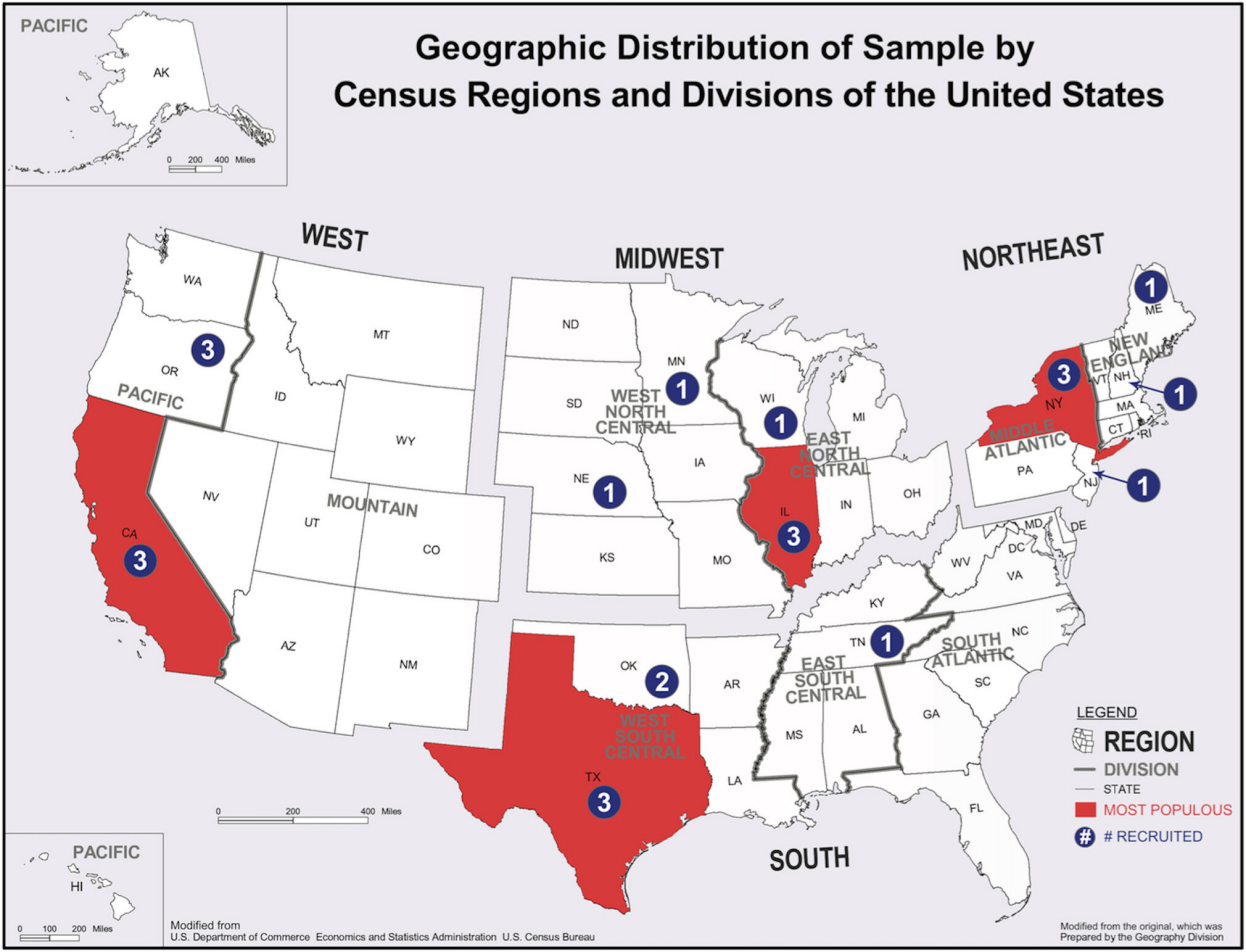
Geographic distribution of final sample of Group 2 organizations by census regions and divisions of the United States (numbers indicate number of organizations sampled in each state). Modified from the original prepared by U.S. Census Bureau Geography Division.

To generate the Group 2 sample, the four U.S. Census Regions were used to divide the United States: Northeast, Midwest, South, and West (see Figure 1). To approximate a probability-proportional-to-size sampling frame, the most populous state in each region was included while other states in the region were chosen at random. Using U.S. Census population figures from 2016, the most populous state in each region was identified (Northeast = New York; Midwest = Illinois; South = Texas; West = California). For the remaining states in the region, each state was assigned a number (1 through x, where x = the number of states in that region). A random number generator (14) was then used to select a number corresponding to one state. This process was repeated for each of the four regions.

### Organization Selection and Randomization (Group 2)

Our goal was to survey six organizations from each region: three from the most populous state and one from each of the three randomly-selected states. Because a comprehensive list of U.S. therapy dog organizations does not currently exist, selecting three organizations per state required identifying and compiling a list of the organizations operating in that state^3^. In order to identify an adequate population from which to select our sample, we attempted to locate 10 organizations that met our inclusion/exclusion criteria from the most populous state and an additional 10 from each randomly-selected state. The process used to identify programs was as follows:

A basic Google search for [therapy dogs + name of state], e.g., therapy dogs New York, was conducted. The project manager then used the inclusion/exclusion criteria to review the first 100 results. After the first 100 results, a new search term was entered: [animal-assisted therapy + name of state], e.g., animal-assisted therapy New York. The project manager then used the inclusion/exclusion criteria to review the first 100 results. The search was stopped when the project manager had obtained a total of 10 organizations, or had reviewed 100 records from each of the two Google searches describe above, whichever came first.

For the most populous states, it was generally possible to identify 10 organizations (only eight were identified in New York). For the less populous states, it was necessary to the repeat the state and organization selection processes described above until a total of 10 organizations were identified from the randomly-selected states in that region (see Table 1 for beginning population and final sample breakdown by state and region). Organizations within a state or group of states were each assigned a number (generally 1–10, but 1–8 for New York). The same random number generator (14) was used to randomly select three numbers. If an organization did not respond, refused to participate, or was otherwise excluded because of pre-specified exclusion criteria, the organization was replaced using the same random selection process described above (see Figure 1 for geographic distribution of final sample by state and region). A total of 33 organizations were invited to participate and, of those, nine did not complete the survey (1 closed, 1 refused, 7 non-responsive after at least two follow-ups) (response rate: 73%).

**Table 1:** Beginning population and final sample breakdown (most populous states are in bold).

**Region**	**State**	**Population: number of organizations identified**	**Final sample: number of organizations completing survey**
**Group 1:**			
National/Multiregional	Multiple	6	4
**Group 2:**			
Northeast	**New York**	8	3
Northeast	Maine	2	1
Northeast	New Hampshire	4	1
Northeast	New Jersey	4	1
Midwest	**Illinois**	10	3
Midwest	Minnesota	3	1
Midwest	Nebraska	2	1
Midwest	Wisconsin	5	1
South	**Texas**	10	3
South	Tennessee	3	1
South	District of Columbia	1	0
South	Oklahoma	4	2
South	Arkansas	2	0
West	**California**	10	3
West	Wyoming	0	0
West	Colorado	5	0
West	Oregon	4	3
West	Montana	1	0

### Procedures

Data collection for the survey was completed between January and March 2018. An introductory cover letter to was sent via e-mail to organizations selected using the procedures described above. The letter explained the project, provided information about participant confidentiality, described the study incentive being offered ($125 donation to the organization), and included contact information to obtain additional information. Once an organization agreed to participate, its representative was provided with a personalized survey link and a six-digit unique identifier. To protect confidentiality, organizations were identified in the data set using these unique identifiers. At the start of the online survey, participants were once again presented with the project description and a statement on confidentiality. Before proceeding with the survey, participants were required to acknowledge and provide consent to have their organization's information included in the project. Participants were generally given 1 month to complete the survey and at least two follow-up attempts were made for non-responders. An incentive of 125 U.S. Dollars, made payable as a donation to the organization, was offered for completion of the survey. Because the survey did not include requests for personal identifying information, this project was determined to be exempt from review by the University of Pennsylvania Institutional Review Board.

### Survey Instrument

An 89-item web-based survey was created using the Qualtrics application (15). The survey items were derived primarily from guidelines published by Lefebvre et al. (5); the Society for Healthcare Epidemiology of America (SHEA) (6, 7); the American Veterinary Medical Association (AVMA) (8), and the Tufts Institute for Human-Animal Interaction (4). A summary of these guidelines and their recommendations is provided in Table S1. Some additional survey items were proposed by members of the advisory panel. An iterative process was employed to create and refine the survey, with each iteration being reviewed by the advisory panel made up of experts from the fields of animal-assisted therapy, animal behavior, animal welfare, human-animal interaction, psychology, research design, social work, and veterinary medicine (see Acknowledgments for a full list of advisory panel members). To reduce respondent burden, the majority of questions were written in a multiple-choice format (e.g., yes, no, unsure, no response/not applicable). The survey was divided into five sections based on item content (see Table 2). After each section, space for optional comments was provided. Skip-logic was employed to ensure that respondents were only presented with items that were relevant based on their previous responses (in the counts below, multi-part questions are counted as one item).

**Table 2:** Survey topics and number of associated items asked of selected therapy dog organizations.

**Survey section**	**Number of items**	**Description of topics covered**
Initial screening questions (Group 2 organizations only)	6	Screening for inclusion/exclusion criteria
Organizational information	5	Organization demographics: Year founded; geographic regions served; number of employees; species utilized; and non-profit status
Registration statistics	7	Number of handlers registered; the typical number of new handlers who apply annually; the failure/rejection rate of new applicants; and the most common reasons that applicants fail or are rejected
Organizational standards and requirements	61	Organizational screening requirements for dogsStandards and practices relating to: Animal welfare; animal behavior, human and animal health; human and animal safetyThe existence of formal written guidelines relating to: Dog requirements; handler requirements; training methods; and reporting incidents such as injuries, damage to property, or misconductThe provision of training or information related to: Canine body language, safeguarding canine welfare, patient confidentiality, reporting adverse incidents, hand hygiene, and zoonosesThe use of internal or external evaluators and trainers to assess and train dogs and handlers Liability insurance requirements
Education and training	10	Required and optional education and/or training for dogs, handlers, evaluators, and trainers Credential requirements for evaluators and trainers

### Statistical Analyses

Fisher Exact and Chi Square Goodness-of-Fit tests were used to investigate differences in the distribution of survey responses between Group 1 and Group 2 organizations. All analyses were performed using JMP Pro 15 statistical software (SAS Institute Inc., 2019).

## Results

### Organizational Demographics

Four of the six Group 1 organizations (67%), and 24 of the 33 (73%) Group 2 organizations completed the survey. The average age of the Group 1 organizations surveyed was 32 years (range: 25–41 years, median = 31 years), and of Group 2 organizations it was 15 years (range: 2–31 years, median = 14.5 years). All Group 1 and 83% of Group 2 organizations were registered as tax-exempt non-profit 501(c) (3) organizations with the U.S. Internal Revenue Service. Group 1 organizations had anywhere from 2 to 17 full- or part-time paid employees (median = 0). While the majority of Group 2 organizations had no paid employees (16), five had between 1 and 3 paid employees, and two had between 10 and 20. Seventy-five percent of Group 1 organizations registered other kinds of therapy animal (principally cats and/or rabbits) besides dogs, while 33% of Group 2 organizations did so.

### Differences Between Group 1 and Group 2 Organizations

Only one significant difference in the distribution of survey responses between Group 1 and Group 2 organizations was noted: Group 2 organizations were significantly more likely to require dogs to be on continuous flea and tick preventative treatment than Group 1 organizations, none of which imposed this requirement (Pearson Chi Square 4.36, *P* = 0.0368). This difference failed to reach significance on the Fisher Exact test, due to the small sample size of Group 1 organizations (*N* = 4).

### Registration Statistics

**Figure 2:**
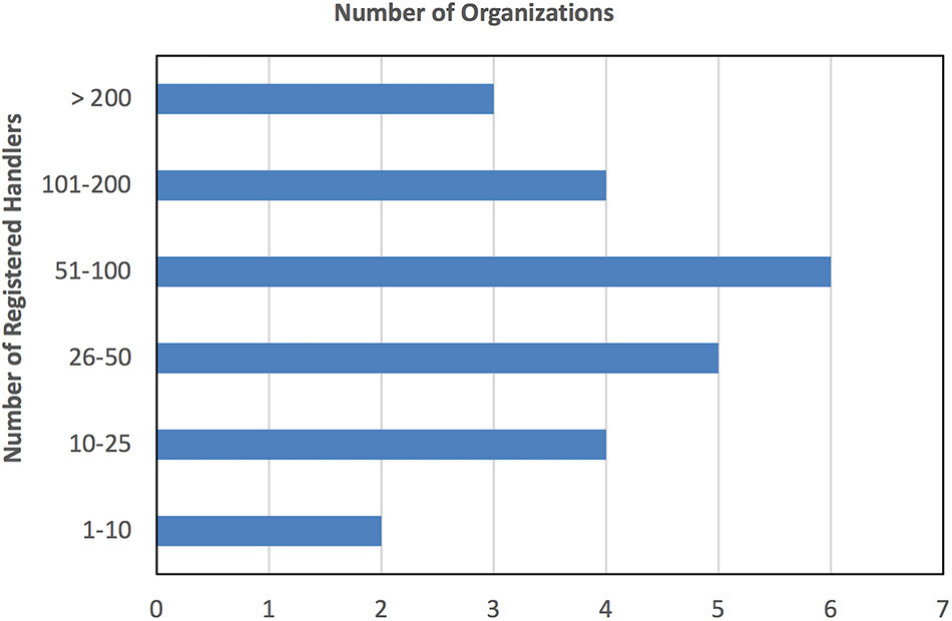
Number of currently-registered handlers in Group 2.

There was substantial variation in the number of handlers registered. All of the Group 1 organizations had more than 200 currently-registered handlers (range: 344–15,000, median = 6,517). Among Group 2, two organizations registered ten or fewer handlers, and three organizations reporting more than 200 (the highest reporting 700; Figure 2).

**Figure 3:**
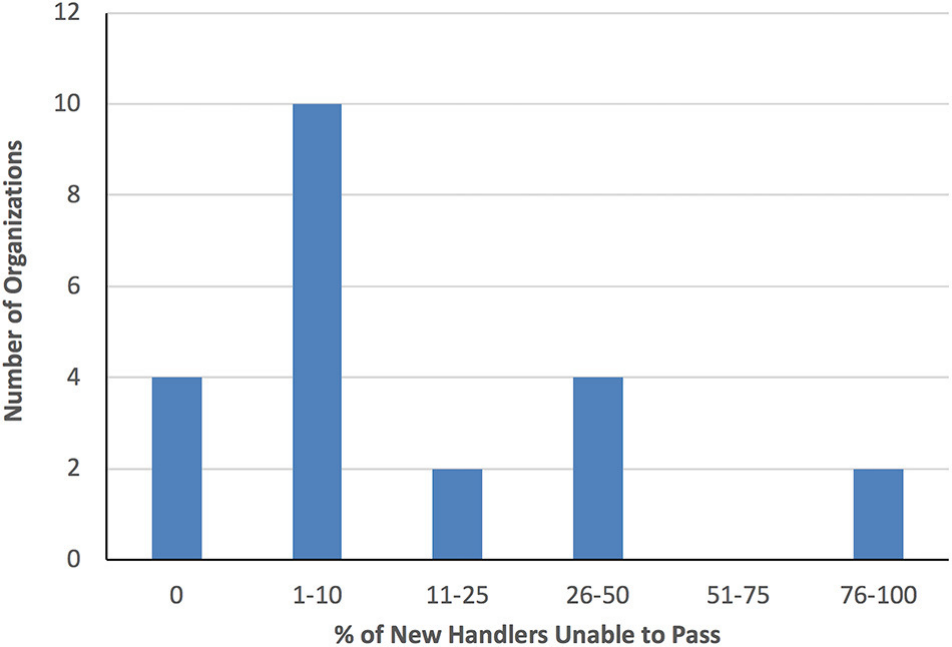
Percentage of new handler applicants who were unable to pass or complete Group 2 organizations' screening process in 2017.

With respect to the survey question regarding the percentage of new applicants who were unable to pass the organization's screening process and register successfully in 2017, the majority of Group 2 organizations reported a failure rate of 10% or less (71%), with six organizations (25%) indicating that all applicants were successful, and 11 organizations (46%) reporting that between 1 and 10% were unsuccessful (Figure 3). Failure rates among Group 1 organizations ranged from 0 to 30%. There was no evidence of association between the per cent of unsuccessful applicants and the total number of handlers registered. For example, among the three Group 2 organizations with more than 200 currently-registered handlers, two reported failing <10% of applicants while the third reported failing 80% of new applicants. The majority of organizations (50% Group 1, 79% Group 2) indicated that dog behavior was the most common reason for an application to be rejected. Fifty percent of Group 1 and 58% of Group 2 organizations keep records of the reason(s) applications are rejected.

### Dog Requirements and Screening Standards

One hundred percent of Group 1 organizations had formal guidelines on dog requirements, required in-person behavioral evaluations, required dogs to be at least 1-year old, and did not require AKC Canine Good Citizen (CGC) certification or spaying/neutering. Lack of consensus among Group 1 organizations was reported with respect to the need for regular behavioral re-evaluation, dogs being in a permanent home for 6 months, and the exclusion of certain breeds from being registered as therapy dogs (Table 3).

**Table 3:** Dog requirements and screening standards in Group 1 (*n* = 4) and Group 2 (*n* = 24) therapy dog organizations.

	**Group 2**			**Group 1**
**Dog requirements and screening standards**	**Yes (%)**	**No (%)**	**Other (%)**	**Yes/no**
Have formal, written guidelines for dog requirements	23 (96)	1 (4)	0	4/0
Require a formal (in-person) behavioral evaluation	22 (92)	2 (8)	0	4/0
Require regular behavioral re-evaluation	14 (58)	8 (33)	2 (8)	2/2
Require AKC CGC certification	9 (38)	15 (63)	0	0/4
Require dog to be at least 1-year of age	20 (83)	3 (13)	1 (4)	4/0
Require dog to be in a permanent home for at least 6 months	11 (46)	12 (50)	1 (4)	2/2
Require dog to be spayed or neutered	5 (21)	19 (79)	0	0/4
Prohibit dogs of certain breeds from being registered	1 (4)	22 (92)	1 (4)	1/3

The majority (96%) of Group 2 organizations reported having formal, written guidelines for what is required for a dog to become successfully registered, 92% require a formal, in-person behavioral evaluation of the dog prior to certification, and 83% require that the dog be at least 1-year old to participate in visits. There is less agreement between Group 2 organizations concerning the need for dogs to have their behavior re-evaluated on a regular basis (58% agree), to have AKC CGC certification (38% agree), or to have lived in their current home for at least 6 months (46% agree). A minority of organizations (26%) required dogs to be spayed or neutered before participating in AAAs, and only 4% prohibited registration of certain breeds of dog (e.g., American Staffordshire terrier, Doberman pinscher; Table 3).

### Dog Health and Safety Standards

Group 1 organizations were consistent in their requirement of rabies vaccinations, avoiding AAAs if dogs are showing signs of poor health (e.g., vomiting, diarrhea, lethargy, etc.), having dogs' nails clipped to a safe length, and keeping dogs leashed at all times, but were less consistent on other standards, with only 25% prohibiting raw meat diets and treats, requiring vaccinations other than rabies, and requiring negative heartworm test results (Table 4).

**Table 4:** Dog health and safety standards in Group 1 (*n* = 4) and Group 2 (*n* = 24) therapy dog organizations.

	**Group 2**			**Group 1**
**Dog health and safety standards**	**Yes (%)**	**No (%)**	**Other (%)**	**Yes/no**
Health clearance from a veterinarian (with documentation)	21 (88)	3 (13)	0	3/1
Physical health re-evaluated by a veterinarian on a regular basis	21 (88)	2 (8)	1 (4)	3/1
Rabies vaccinations (with documentation)	21 (88)	2 (8)	1 (4)	4/0
Distemper/adenovirus/parvovirus vaccinations (with documentation)	15 (63)	8 (33)	1 (4)	1/3
Leptospirosis vaccinations (with documentation)	7 (29)	14 (58)	3 (13)	1/3
Bordetella vaccinations (with documentation)	7 (29)	15 (63)	2 (8)	1/3
Canine influenza vaccinations (with documentation)	5 (21)	17 (71)	2 (8)	0/4
Other vaccinations (not specified above)	3 (13)	15 (63)	6 (25)	0/4
Negative fecal parasite results	18 (75)	3 (13)	3 (13)	3/1
Negative heartworm results	9 (38)	12 (50)	3 (13)	1/3
Continuous flea/tick preventative	13 (54)	10 (42)	1 (4)	0/4
Not currently taking immunosuppressive medications or antibiotics	8 (33)	12 (50)	4 (17)	3/1
Avoid AAAs if showing signs of poor health (e.g., lethargy, diarrhea, vomiting)	22 (92)	0 (0)	2 (8)	4/0
Avoid raw meat diets and treats	3 (13)	18 (75)	3 (13)	1/3
Bathed within 24 h of visits	10 (42)	9 (38)	5 (21)	2/2
Nails clipped to safe length prior to visits	17 (71)	5 (21)	2 (8)	4/0
Require dog to be leashed at all times	23 (96)	1 (4)	0	4/0
Allow handlers to bring >1 dog per visit	4 (17)	20 (83)	0	0/4

Of the Group 2 organizations surveyed, 96% require dogs to be leashed at all times during visits, 92% prohibit the use of dogs showing signs of poor health, while 88% require that participating dogs receive health clearance from a veterinarian, and mandate re-evaluation by a veterinarian on a regular basis. Eighty-eight percent require that dogs be vaccinated for rabies, and 63% require vaccination for distemper/adenovirus/parvovirus. Eighty-three percent disallow handlers from visiting with more than one dog at a time, 75% require a negative fecal parasite test result, and 54% require continuous flea/tick preventative treatment. Conversely, only 42% of Group 2 organizations require that dogs be bathed within 24 h prior to visits, 33% disallow dogs from visiting when currently taking immunosuppressive medications or antibiotics, 13–29% require vaccinations against other infections, and 13% prohibit the feeding of raw meat diets and treats (Table 4).

### Dog Welfare Standards

Only two of the four Group 1 organizations imposed time limits on the length of visits, ranging from 1 to 2 h (Table 5). Seventy-five percent had formal policies on acceptable training methods and provide training/information on body language and canine welfare.

**Table 5:** Dog welfare standards for Group 1 (*n* = 4) and Group 2 (*n* = 24) therapy dog organizations.

	**Group 2**			**Group 1**
**Dog welfare standards**	**Yes (%)**	**No (%)**	**Other (%)**	**Yes/No**
Limit the length of time (per visit) that dogs may work	12 (50)	10 (42)	2 (8)	2/2
Have formal (written) policy on acceptable training methods	18 (75)	5 (21)	1 (4)	3/1
• Prohibit use of prong, choke, e-collars, etc.	11 (60)	7 (40)	0	3/1
• Require use of positive reinforcement training	11 (60)	7 (40)	0	3/1
• Both	6 (32)	12 (68)	0	1/3
Provide training/information on canine body language	21 (88)	3 (12)	0	3/1
Provide training/information on safeguarding canine welfare	23 (96)	1 (4)	0	3/1

**Figure 4:**
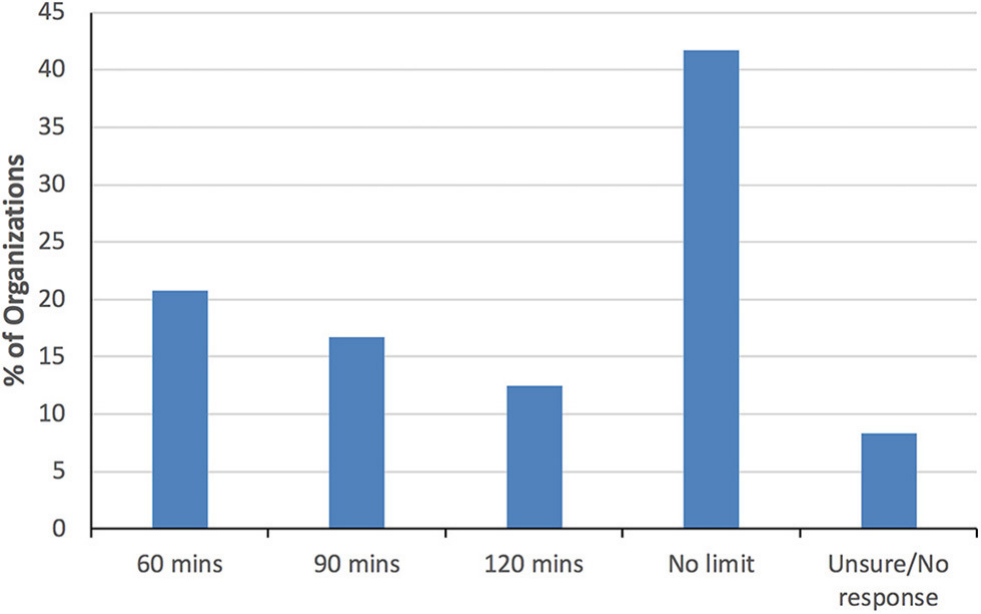
Group 2 organizational limits on the duration of animal-assisted intervention visits by therapy dogs.

Of the 24 Group 2 organizations that responded, the majority provide training and/or information on safeguarding canine welfare (96%) and reading canine body language (88%). Despite the provision of such information, only 50% indicated that they imposed limits on the duration of AAI visits, and when limits were imposed, they ranged from 1 to 2 h (Figure 4). Seventy-five percent reported having formal (written) policies on acceptable/unacceptable training methods for use with therapy dogs. When asked to specify briefly the training methods that are/are not permitted, 60% of organizations reported disallowing the use of coercive training equipment (e.g., prong collars, choke collars, e-collars, etc.), 60% required the exclusive use of positive reinforcement (rewards-based) training, and 32% stipulated both (Table 5).

The pattern of dog welfare standards for Group 1 organizations were comparable to those in Group 2. Of the two organizations that imposed time limits on the length of visits, the range was again from 1 to 2 h (Table 5).

### Handler Health and Safety Standards

All Group 1 organizations require the handler to avoid visits if symptoms of communicable disease (e.g., fever, cough, diarrhea, etc.) are present, but only 33% require immunizations recommended for healthcare workers, that handlers be at least 18 years of age, and that handlers receive criminal background checks and child abuse history clearance. None of the Group 1 organizations require handlers to receive health screening from their physician.

The majority of Group 2 organizations (92%) discourage volunteer handlers from making visits when displaying symptoms of communicable disease. However, only 50% recommend that handlers receive appropriate immunizations, or avoid visits if other members of their household have symptoms of communicable illness. Routine health screening of handlers by their physicians is recommended by only a minority of organizations (25%). Criminal background checks and child abuse history clearances (if working with minors) are required by 54 and 42% of Group 2 organizations, respectively, and 58% require that therapy dog handlers be at least 18 years of age (Table 6).

**Table 6:** Handler health and safety standards for Group 1 (*n* = 4) and Group 2 (*n* = 24) therapy dog organizations.

	**Group 2**			**Group 1**
**Handler health and safety standards**	**Yes (%)**	**No (%)**	**Other (%)**	**Yes/no**
Receive health screening from their physician (with documentation)	6 (25)	15 (63)	3 (13)	0/4
Receive immunizations recommended for healthcare providers (if visiting healthcare facilities)	12 (50)	10 (42)	2 (8)	1/3
Avoid visits if symptoms of communicable illnesses present (e.g., fever, cough, diarrhea)	22 (92)	1 (4)	1 (4)	4/0
Avoid visits if other members of the household have symptoms of communicable illnesses	12 (50)	10 (42)	2 (8)	3/1
Be at least 18 years of age	14 (58)	10 (42)	0	1/3
Receive child abuse history clearances (if working with minors)	10 (42)	9 (38)	5 (21)	1/3
Receive criminal background checks	13 (54)	8 (33)	3 (13)	1/3

### Handler Training/Education Standards

Among Group 1 organizations, 75% require handler training, shadowed visits, and training on zoonotic diseases and patient confidentiality. However, only 50% require training on hand hygiene or cleaning animal waste.

Seventy-nine percent of the Group 2 organizations that participated require volunteer therapy dog handlers to undergo training before starting AAA visits, and the same proportion provide training or information on maintaining patient confidentiality. Eighty-three percent offer training or information about how to report adverse events/incidents, and 88% require that handlers be shadowed on at least one AAA visit before being allowed to visit independently. A smaller proportion of these organizations provide training/information on hand hygiene (63%), cleaning up animal waste (58%), and zoonotic disease transmission (46%; Table 7).

**Table 7:** Handler training/education standards for Group 1 (*n* = 4) and Group 2 (*n* = 24) therapy dog organizations.

	**Group 2**			**Group 1**
**Handler training/education standards**	**Yes (%)**	**No (%)**	**Other (%)**	**Yes/no**
Handlers are required to participate in training before starting AAA visits	19 (79)	4 (17)	1 (4)	3/1
Handlers must be 'shadowed' on AAA visits before they can visit independently	21 (88)	3 (13)	0	3/1
Receive training or information on zoonotic disease transmission	11 (46)	10 (42)	3 (13)	3/1
Receive training or information on hand hygiene (i.e., hand washing)	15 (63)	7 (29)	2 (8)	2/2
Receive training or information on cleaning animal waste	14 (58)	8 (33)	2 (8)	2/2
Receive training or information on maintaining patient confidentiality	19 (79)	4 (17)	1 (4)	3/1
Receive training or information about how to report incidents	20 (83)	3 (13)	1 (4)	4/0

## Discussion

Despite broad areas of correspondence between the current findings and the recommendations of published guidelines (see Table S1), there are also numerous areas where these recommendations have not been absorbed or adopted widely by the therapy dog organizations that participated in the current survey. Some of these discrepancies may be relatively inconsequential, but others have the potential to give rise to unacceptable and largely preventable risks to the health, safety, and welfare of AAI recipients and/or the dogs who provide this service.

### Dog Requirements and Screening

Most of the current guidelines for facilities require that dogs be formally evaluated for suitable behavior and temperament using tests designed to simulate the circumstances they might encounter in hospital settings. They also require that dogs be re-evaluated at least every 2–3 years, that they be at least 1-year of age, and have lived with their current handlers for a minimum of 6 months before participating in visits. None of the existing guidelines requires or advocates AKC CGC certification for therapy dogs, or specifically opposes the participation of particular breeds of dog, or of sexually intact dogs, although most recommend the exclusion of intact female dogs when “in heat” (4, 7, 8).

Of the organizations surveyed in the current study, the majority were compliant with the requirement for initial temperament/behavioral evaluations of each dog, and that dogs participating in AAIs be at least 1-year of age. Most did not require therapy dogs to be spayed/neutered, to be AKC CGC certified, nor did they prohibit the involvement of certain breeds. A substantial proportion of Group 1 and 2 organizations, however, did not require periodic behavioral re-evaluations of therapy dogs, or restrict registration to dogs that had lived in their current homes for a minimum of 6 months, despite these being widely recommended by existing guidelines. Although the original Lefebvre et al. (5) committee reached consensus on the need for behavioral re-evaluation, they also acknowledged that this was an “unresolved issue” in the sense that there was no empirical or epidemiological evidence to support the recommendation. In light of this uncertainty, it would be constructive to examine the records of organizations that currently conduct re-evaluations to determine if the frequency of dogs failing their re-evaluations is sufficient to justify this requirement. Similarly, a comparative assessment of adverse incidents involving dogs that either have or have not lived with their handlers for 6 months might help to determine whether this restriction is also warranted.

### Dog Health and Safety

In accordance with existing facility guidelines, nearly all of the organizations surveyed required dogs to be leashed at all times during visits and to avoid visits when exhibiting signs of ill health (e.g., lethargy, diarrhea, vomiting, etc.). Most (88%) also require documented health clearance from a veterinarian before visiting, and regular veterinary re-evaluations. Similarly, 83% of Group 2, and all Group 1 organizations, disallow visits involving more than one dog per handler. The majority of organizations required that dogs be vaccinated against rabies but there was less agreement regarding the need for immunization against canine distemper/adenovirus/ parvovirus, leptospirosis, *Bordetella*, influenza, and other unspecified pathogens. Rabies vaccination is mandated by state law (17), while the additional vaccinations are administered primarily to prevent the spread of disease between dogs. Leptospirosis and *Bordetella* are zoonotic diseases that can be passed from animals to humans. Therefore, it may be prudent to recommend these for therapy dogs on an *as needed* basis where these diseases are endemic to the area (16).

While most of the published guidelines agree that animals taking immunosuppressive medications and/or antibiotics should potentially be excluded from participating in AAIs, three fourths of Group 1 and only 33% of Group 2 organizations reported this as a restriction. According to Murthy et al. (6):

“*Animals with other concerning medical conditions should be excluded from visitation until clinically normal (or the condition is managed such that the veterinarian feels that it poses no increased risk to patients) and have received a written veterinary health clearance. Examples include episodes of vomiting or diarrhea; urinary or fecal incontinence; episodes of sneezing or coughing of unknown or suspected infectious origin; animals currently on treatment with non-topical antimicrobials or with any immunosuppressive medications.”*

The feeding of raw meat diets to dogs is controversial for a number reasons, one of which is the potential transmission of infectious pathogens to humans (18–21). All of the facility guidelines specify that animals being fed raw meat diets and treats should be excluded from participating in AAIs, but only 13% of the Group 2 organizations surveyed reported this as a restriction—somewhat fewer than the 19% reported previously by Linder et al. (11)—and only one of the Group 1 organizations did so. This finding is highly concerning. Studies have shown that up to 48% of raw meat diets are contaminated with *Salmonella* spp., 20% with *Clostridium* and *Listeria* spp., and a recent report showed transmission of tuberculosis due to a commercial raw meat diet. All of this highlights the potential risk of transmission of infectious pathogens to humans from therapy animals eating these diets. It is unclear why so many organizations fail to comply with this recommendation given the established risks of zoonotic disease transmission, particularly for immunocompromised patients. Despite the lack of supporting evidence, advocates of raw meat diets for dogs are apparently convinced of their health benefits (22). The popularity of dehydrated—but otherwise raw—treats for dogs, and the difficulty of enforcing this restriction, are also likely to be factors. Anecdotally, some organizations appear to be unaware of this issue while others ignore the concerns. Similarly, very few healthcare or eldercare facilities know to ask visiting therapy animal organizations or individual handlers if raw meat diets or treats are allowed. Further research on the prevalence of zoonotic pathogens among dogs associated with organizations that do and do not impose this restriction would be instructive.

All of Group 1 and 71% of Group 2 organizations were compliant with the recommendation that dogs' nails be clipped to a safe length prior to visits, but there was less agreement regarding the need to bathe dogs within 24 h prior to visits. There is also some differences of opinion among the existing guidelines on this point. Whereas, all of the guidelines highlight the importance of attending to the animal's hair coat and skin condition, only one advocates bathing dogs within 24 h of visitation (4), while another specifically warns against the dangers of excessive bathing of therapy dogs (8). The rest recommend brushing or combing dogs prior to visits, but advocate bathing them only when the animal is malodorous or visibly soiled (5, 6). Furthermore, evidence of methicillin-resistant *Staphylococcus aureus* and *Clostridioides difficile* acquisition among therapy dogs visiting healthcare facilities suggests that hygienic measures may be just as important immediately after and between such visits as before them (23, 24). Again, additional research on this topic would be useful, but these results highlight the importance of educating facilities on questions they should be asking before any therapy dog visits (4).

### Dog Welfare

Encouragingly, the majority of Group 2 organizations—both large and small—reported providing their volunteer handlers with information and training on canine welfare (96%) and body language (88%). Somewhat surprisingly, one of the four larger national/multiregional organizations (Group 1) provided no training on these topics. For obvious reasons, dogs and other therapy animals cannot provide “informed consent” to participate in AAIs, but they are quite capable of signaling assent and dissent through their actions and behavior before, during, and after therapy sessions. Providing their handlers with practical knowledge on how to recognize and act upon these behavioral indicators of stress/distress would appear to be a minimum requirement toward safeguarding the welfare of these animals.

Despite recommendations, both in the literature and in guidelines [(1, 12, 25, 26); Table S1], half of Group 1 and 42% of the Group 2 organizations imposed no formal limits on the duration of working visits, but instead left the decision to the owner/handler. Furthermore, many of those that did impose limits tended to be more permissive of longer visits (1.5–2 h) than most of the published guidelines, which generally recommend 1 h or less. Unfortunately, reliable scientific evidence concerning safe time limits for therapy dog visits is sparse, and presumably the associated stress will vary depending on the quality, emotional intensity, and frequency of visits, the training and experience of handlers, and the age, temperament, and perhaps breed of individual dogs (1, 27–30). Most experts agree, however, that such visits have the potential to be stressful for some dogs (12, 31–33), and this suggests that the industry should err on the side of shorter rather than longer visits. Further research on how therapy dogs respond behaviorally and physiologically to visits of different lengths, frequencies, and intensities would help to provide empirical guidance on this issue in the future.

While 75% of organizations in both groups had formal written policies on acceptable/unacceptable training methods for use with therapy dogs, a surprising number of Group 1 and 2 organizations (25 and 40%, respectively) did not explicitly disallow coercive training aids (e.g., choke collars, prong collars, e-collars, etc.) or the use of positive punishment by handlers. While the lack of any formal prohibition does not necessarily imply that therapy dog handlers are using aversive training methods in practice, it is concerning that such a large proportion of these ostensibly dog-friendly organizations are failing to require humane handling and training by volunteer handlers, given abundant evidence of the extent to which aversive or punishment-based training can harm canine welfare and learning ability, as well as the owner-dog bond (34–37).

Though not addressed in the current survey, the question of when and how to retire a therapy animal is clearly also relevant to welfare, and should be included in any future discussions of policy and practice standards within the industry (38).

### Handler Health and Safety

In agreement with all of the facility guidelines, the vast majority of organizations surveyed (100% of Group 1; 92% of Group 2) required that therapy dog handlers refrain from visiting when they themselves are exhibiting symptoms of communicable diseases (e.g., fever, cough, diarrhea, etc.). Compliance with other recommended aspects of handler health and safety was more variable. Only 50% of Group 2 organizations require handlers to refrain from visits when other members of their households are displaying disease symptoms, and that handlers be appropriately immunized before visiting healthcare facilities. Only a minority of organizations (0% of Group 1; 25% of Group 2) require that handlers receive health screening from their physician before participating in AAIs. There was also a general lack of consensus regarding the need for handlers to be over 18 years of age, to receive criminal background checks, or to have child abuse history clearances (if visiting minors). These areas of disagreement within the industry would probably benefit from further discussion and scrutiny.

### Handler Training and Education

The majority of organizations surveyed required their dog handlers to participate in some form of training before being allowed to conduct visits to healthcare facilities, and an even higher proportion require that handlers be observed on at least one visit before being allowed to visit independently. With respect to types of instruction, most organizations provide training or information on how to report adverse incidents and maintain patient confidentiality, but substantially fewer provided instruction on basic hygiene, cleaning animal waste, or zoonotic disease transmission.

Finally, the study has some important limitations that should be noted. The organizations that responded to the current survey represent a relatively small fraction of the total number of therapy animal organizations that exist nationally. While every effort was made to select a representative sample of organizations to participate in the survey, it remains possible that a larger sample of responding organizations would have generated different findings. In addition, survey data have certain inherent limitations. The questionnaire items employed in the current survey were tested for face validity by a panel of experts, many of whom were members of therapy animal organizations. However, the items were not formally pre-tested for reliability or validity on a pilot sample of organizations, and this raises some doubts about the consistency of responses, and whether the items actually measure what they were intended to measure or evaluate. Future studies on this topic should aim to avoid these shortcomings.

## Summary and Conclusions

In summary, to the extent that the sample of therapy dog organizations that contributed to this study is representative of the country as a whole, the current findings have identified several areas in which a substantial proportion of organizations depart from what would generally be considered “best practice” as defined by existing guidelines [(4–8); Table S1]. With respect to animal welfare, it is concerning that so many organizations impose no formal time limits on the length of visits, or impose limits that exceed those recommended by most of the guidelines. The lack of consensus on the need for strictly reward-based, positive reinforcement training and control methods for therapy dogs is also a source of concern. Most alarming from a risk management and infection control perspective were the findings that only a minority of organizations imposed restrictions on visits by dogs fed raw meat diets or treats, or those currently taking antibiotic or immunosuppressive medications, and that few provided their handlers with any information or instruction on zoonotic disease transmission or prevention. Organizations were also inconsistent regarding the need for dogs to be regularly re-evaluated for changes in behavior/temperament, vaccinated against Leptospirosis and *Bordetella*, have their nails clipped to a safe length, have continuous flea/tick preventative treatment, and that handlers should be appropriately immunized (e.g., against influenza), be at least 18 years old, subject to criminal background checks and child abuse history clearances (when working with minors), receive training/information on hand hygiene and cleaning animal waste, and avoid visits when other members of their households are showing symptoms of communicable disease.

These results highlight many areas in need of further study, as well as the need for facilities to be aware of the wide discrepancies among therapy animal organizations' requirements. This emphasizes the importance of facilities asking questions to ensure that therapy dog organizations and individual therapy animal teams meet appropriate standards to protect human and canine health and to ensure animal welfare. Hopefully, these results will help stimulate constructive debate leading toward the goal of an industry-wide consensus on both minimum acceptable and ideal standards to ensure the health, welfare, and safety of both human and animal participants in AAIs.

## Data Availability Statement

The datasets generated for this study are available on request to the corresponding author.

## Author Contributions

JS and KK designed and conducted the study and wrote the paper. LF, JG, and ZN contributed substantially to survey development, sample recruitment, and critical review of the manuscript.

### Conflict of Interest

The authors declare that the study received funding from Mars Petcare and the WALTHAM Center for Pet Nutrition. The funders were not involved in the study design, collection, analysis, interpretation of the data, the writing of this article or the decision to submit it for publication. While conducting the study, JS served as a paid consultant to Mars Inc. on issues unrelated to the submitted work.

## Footnotes

^1^Alliance of Therapy Dogs (formerly Therapy Dogs, Inc.), Bright and Beautiful Therapy Dogs, Intermountain Therapy Animals, Love on a Leash, Pet Partners (formerly Delta Society), and Therapy Dogs International (TDI).

^2^Organizations were not excluded if they required that all handlers be registered with one of the six largest/most prominent certifying organizations but did not stipulate which one. The rationale behind this decision was that, in these cases, the organization was unlikely to be following one uniform set of guidelines from one particular organization.

^3^An organization had to have its main office/mailing address in a particular state to be counted as belonging to that state.

## References

[1] Ng ZY Advocacy and rethinking our relationships with animals: ethical responsibilities and competencies in animal-assisted interventions. In: Tedeschi P Jenkins MA, editors. Transforming Trauma: Resilience and Healing Through Our Connections with Animals. West Lafayette, IN: Purdue University Press (2019).

[2] AKC AKC Recognized Therapy Dog Organizations. Available online at: https://www.akc.org/sports/title-recognition-program/therapy-dog-program/therapy-dog-organizations (accessed August 20, 2019).

[3] Hardin P Brown J Wright ME. Prevention of transmitted infections in a pet therapy program: an exemplar. Am J Infect Control. (2016) 44:846–50. 10.1016/j.ajic.2016.01.007 27372389

[4] Freeman L Linder D Mueller M Gibbs D Animal-Assisted Interventions: How-to Guide for Facilities. North Grafton, MA: Tufts Institute for Human-Animal Interaction (2016). p. 1–29.

[5] Lefebvre SL Golab GC Christensen E Castrodale L Aureden K Bialachowski A. Guidelines for animal-assisted interventions in health care facilities. Am J Infect Control. (2008) 36:78–85. 10.1016/j.ajic.2007.09.005 18313508

[6] Murthy R Bearman G Brown S Bryant K Chinn R Hewlett A. Animals in healthcare facilities: recommendations to minimize potential risks. Infect Control Hosp Epidemiol. (2015) 36:495–516. 10.1017/ice.2015.15 25998315

[7] The Society for Healthcare Epidemiology of America Animals in Healthcare Facilities. (2015). Available online at: https://www.guidelinecentral.com/ (accessed October 15, 2017).

[8] American Veterinary Medical Association Animal-Assisted Interventions: Guidelines: American Veterinary Medical Association. (n.d.) Available online at: https://www.avma.org/KB/Policies/Pages/Animal-Assisted-Interventions-Guidelines.aspx (accessed November 28, 2017).

[9] Delta Society Standards of Practice for Animal-assisted Activities and Animal-assisted Therapy. Renton, WA: Delta Society (1996).

[10] Hines L Fredrickson M Perspectives on animal-assisted activities and therapy. In: Wilson CC Turner DC, editors. Companion Animals in Human Health. Thousand Oaks, CA: Sage Publications (1998). p. 23–39. 10.4135/9781452232959.n2

[11] Linder DE Siebens HC Mueller MK Gibbs DM Freeman LM. Animal-assisted interventions: a national survey of health and safety policies in hospitals, eldercare facilities, and therapy animal organizations. Am J Infect Control. (2017) 45:883–7. 10.1016/j.ajic.2017.04.287 28673680 PMC5542869

[12] Serpell JA Coppinger R Fine AH Peralta JM Welfare consideration in therapy and assistance animals. In: Fine AH, editor. Handbook on Animal-Assisted Therapy: Theoretical Foundations and Guidelines for Practice. 3rd ed San Diego, CA: Academic Press (2010). p. 481–503. 10.1016/B978-0-12-381453-1.10023-6

[13] Serpell J McCune S Gee N Griffin JA Current challenges to research on animal-assisted interventions. Appl Develop Sci. (2017) 21:223–33. 10.1080/10888691.2016.1262775

[14] Urbaniak GC Plous S Research Randomizer. (2013) Available online at: https://www.randomizer.org/ (accessed August 20, 2019).

[15] Qualtrics Qualtrics. Provo, UT (2018).

[16] Ford RB Larson LJ Schultz RD Welborn LV. 2017 AAHA Canine vaccination guidelines. Am Anim Hosp Assoc. (2017) 53:243–51. 10.5326/JAAHA-MS-6741 28846453

[17] Brown CM Slavinski S Ettestad P Sidwa TJ Sorhage FE. Compendium of animal rabies prevention and control, 2016. J Am Vet Med Assoc. (2016) 248:505–17. 10.2460/javma.248.5.505 26885593

[18] Lefebvre SL Reid-Smith R Boerlin P Weese JS. Evaluation of the risks of shedding salmonellae and other potential pathogens by therapy dogs fed raw diets in Ontario and Alberta. Zoonoses Public Health. (2008) 55:470–80. 10.1111/j.1863-2378.2008.01145.x 18811908

[19] Freeman LM Chandler ML Hamper BA Weeth LP. Current knowledge about the risks and benefits of raw meat–based diets for dogs and cats. J Am Vet Med Assoc. (2013) 243:1549–58. 10.2460/javma.243.11.1549 24261804

[20] Davies RH Lawes JR Wales AD. Raw diets for dogs and cats: a review, with particular reference to microbiological hazards. J Small Anim Pract. (2019) 60:329–39. 10.1111/jsap.13000 31025713 PMC6849757

[21] O'Halloran C Ioannidi O Reed N Murtagh K Dettemering E Poucke SV. Tuberculosis due to *Mycobacterium bovis* in pet cats associated with raw food diet. J Feline Med Surg. (2019) 21:667–81. 10.1177/1098612X19848455 31082328 PMC10814295

[22] Morelli G Bastianello S Catellani P Ricci R. Raw meat-based diets for dogs: survey of owners' motivations, attitudes and practices. BMC Vet Res. (2019) 15:74. 10.1186/s12917-019-1824-x 30832667 PMC6399943

[23] Enoch DA Karas JA Slater JD Emery MM Kearns AM Farrington M. MRSA carriage in a pet therapy dog. J Hosp Infect. (2005) 60:186–8. 10.1016/j.jhin.2004.11.011 15866022

[24] Lefebvre SL Reid-Smith RJ Waltner-Toews D Weese JS. Incidence of acquisition of methicillin-resistant *Staphylococcus aureus, Clostridium difficile*, and other health-care–associated pathogens by dogs that participate in animal-assisted interventions. J Am Vet Med Assoc. (2009) 234:1404–17. 10.2460/javma.234.11.1404 19480620

[25] Iannuzzi D Rowan AN Ethical issues in animal-assisted therapy programs. Anthrozoös. (2015) 4:154–63. 10.2752/089279391787057116

[26] International Association of Human-Animal Interaction Organizations The Iahaio Definitions for Animal Assisted Intervention and Guidelines for Wellness of Animals Involved in Aai. Seattle, WA: International Association of Human-Animal Interaction Organizations (2018).

[27] Glenk LM Kothgassner OD Stetina BU Palme R Kepplinger B Baran H Therapy dogs' salivary cortisol levels vary during animal-assisted interventions. Anim Welfare. (2013) 22:369–78. 10.7120/09627286.22.3.369

[28] Hatch A The view from all fours: a look at an animal-assisted activity program from the animal's perspective. Anthrozoös. (2007) 20:37–50. 10.2752/089279307780216632

[29] King C Watters J Mungre S Effect of a time-out session with working animal-assisted therapy dogs. J Vet Behav. (2011) 6:232–8. 10.1016/j.jveb.2011.01.007

[30] Ng ZY Pierce BJ Otto CM Buechner-Maxwell VA Siracusa C Werre SR The effect of dog–human interaction on cortisol and behavior in registered animal-assisted activity dogs. Appl Anim Behav Sci. (2014) 159:69–81. 10.1016/j.applanim.2014.07.009

[31] Butler K Therapy Dogs Today: Their Gifts, Our Obligation. Norman, OK: Funpuddle Publishing (2004).

[32] Haubenhofer DK Kirchengast S. Physiological arousal for companion dogs working with their owners in animal-assisted activities and animal-assisted therapy. J Appl Anim Welf Sci. (2006) 9:165–72. 10.1207/s15327604jaws0902_5 16956319

[33] Mongillo P Pitteri E Adamelli S Bonichini S Farina L Marinelli L Validation of a selection protocol of dogs involved in animal-assisted intervention. J Vet Behav. (2015) 10:103–10. 10.1016/j.jveb.2014.11.005

[34] Deldalle S Gaunet F Effects of 2 training methods on stress-related behaviors of the dog (Canis familiaris) and on the dog–owner relationship. J Vet Behav. (2014) 9:58–65. 10.1016/j.jveb.2013.11.004

[35] Hiby EF Rooney NJ Bradshaw JWS Dog training methods: their use, effectiveness and interaction with behaviour and welfare. Anim Welfare. (2004) 13:63–9.

[36] Reisner I The learning dog: a discussion of training methods. In: Serpell JA, editor. The Domestic Dog: Its Evolution, Behavior and Interactions with People. 2 ed Cambridge: Cambridge University Press (2016). p. 211–26. 10.1017/9781139161800.011

[37] Rooney NJ Cowan S Training methods and owner–dog interactions: links with dog behaviour and learning ability. Appl Anim Behav Sci. (2011) 132:169–77. 10.1016/j.applanim.2011.03.007

[38] Ng ZY Fine AH. Considerations for the retirement of therapy animals. Animals. (2019) 9:110. 10.3390/ani9121100 31835308 PMC6941057

