# Supplementary material for: Current Standards and Practices Within the Therapy Dog Industry: Results of a Representative Survey of United States Therapy Dog Organizations
Source: Front Vet Sci. 2020 Feb 7;7:35. doi: 10.3389/fvets.2020.00035 (PMC7020743; doi:10.3389/fvets.2020.00035)
Supplement: Supplementary file 1 [file Table_1.DOCX]

Table S1: Summary of recommendations of published guidelines for healthcare facilities on animal-assisted interventions

| ***Dog Requirements and Screening Standards*** | ***SHEA (7)*** | ***Lefebvre et al. (5)*** | ***Freeman et al. (4)*** | ***AVMA (15)*** |
| --- | --- | --- | --- | --- |
| *Minimum age of dog* | ≥ 1 year | ≥ 1 year | ≥ 1 year | 6 mos |
| *Animals should be formally evaluated for AAA suitability* | Y | Y | Y |  |
| *Animals should be re-evaluated for AAA suitability at least every:* | 3 years | 3 years | 2 years |  |
| *Dog should pass a temperament evaluation specific to AAAs* | Y | Y |  |  |
| *Dog should be in permanent home ≥ 6 months* | Y | Y |  |  |
| *Dog should have relationship with handler for ≥ 6 months* |  |  | Y |  |
|  |  |  |  |  |
| ***Dog Health and Safety Standards*** | ***SHEA (7)*** | ***Lefebvre et al. (5)*** | ***Freeman et al. (4)*** | ***AVMA (15)*** |
| *Animals should receive health evaluation by veterinarian* | Y | Y | Y | Y |
| *Animals should have their health re-evaluated by a veterinarian* | ≥ 1x/year | ≥ 1x/year | 1x/year | continuous |
| *Require that dogs be vaccinated against rabies* | Y | Y | Y | Y |
| *Rabies titers can be substituted for a vaccine* | N | N |  |  |
| *Internal/external parasite prevention (in accordance with local risks/life stage)* | Y | Y |  | Y |
| *Exclude animals with known or suspected communicable diseases* | Y | Y | Y |  |
| *Potentially exclude animals on immunosuppressants and/or antimicrobials* | Y | Y | Y | Y |
| *Bathing/grooming/hair/skin maintenance* | Y | Y | Y | Y |
| *Nail care (e.g., clipped short and free of sharp edges)* | Y | Y | Y | Y |
| *Keep dog leashed at all times* | Y | Y | Y |  |
| *Limit visits to one animal per handler* | Y | Y | Y |  |
| *Exclude animals that have been fed raw or dehydrated (but otherwise raw) foods* | Y | Y | Y |  |
|  |  |  |  |  |
| ***Dog Welfare Standards*** | ***SHEA (7)*** | ***Lefebvre et al. (5)*** | ***Freeman et al. (4)*** | ***AVMA (15)*** |
| *Handlers should receive training on animal body language* | Y | Y |  | Y |
| *Limit the length of time (per visit) that animals may work* | 60 min | 60 min | 120 min |  |
| *Ensure animals have opportunities for rest* |  |  |  | Y |
| *Exclude the use of choke chains or prong collars* | Y | Y |  |  |
|  |  |  |  |  |
| ***Handler Health and Safety Standards*** | ***SHEA (7)*** | ***Lefebvre et al. (5)*** | ***Freeman et al. (4)*** | ***AVMA (15)*** |
| *Handlers should be formally evaluated* | Y |  | Y |  |
| *Handlers should be appropriately immunized* | Y | Y | Y |  |
| *Handler should refrain from AAA visits when ill (e.g., cough, fever, diarrhea)* | Y | Y | Y |  |
| *Handler should refrain from AAA visits when other people in the house are ill (e.g., cough, fever, diarrhea)* |  |  | Y |  |
| *Handler age should be:* |  |  | >10 10-16 with adult |  |
|  |  |  |  |  |
| ***Handler Training/Education Standards*** | ***SHEA (7)*** | ***Lefebvre et al. (5)*** | ***Freeman et al. (4)*** | ***AVMA (15)*** |
| *Handlers should be formally trained* | Y | Y | Y |  |
| *Animal-handler teams should be observed at least once during visits for being granted approval* | Y | Y |  |  |
| *Handler training should include: Zoonotic diseases* | Y | Y |  |  |
| *Handler training should include: Hand hygiene* | Y | Y |  |  |
| *Handler training should include: Cleaning and disinfecting surfaces contaminated by animal waste* | Y | Y |  |  |
| *Handler training should include: Proper disposal of animal waste* | Y | Y |  |  |
| *Handler training should include: Visual inspection for ectoparasites* | Y | Y |  |  |
| *Handler training should include: Identifying appropriate contacts in the event of an accident or injury* | Y | Y |  |  |
| *Handler training should include: Human Resources policies of facility being visited (e.g., HIPPA)* | Y | Y |  |  |
| *Handler must report any injuries or inappropriate animal behavior* | Y | Y | Y |  |
